# Voluntary, temporary out-of-home firearm storage: a survey of law enforcement agencies in two states

**DOI:** 10.1186/s40621-022-00389-3

**Published:** 2022-07-21

**Authors:** Marian E. Betz, Sara Brandspigel, Leslie M. Barnard, Rachel L. Johnson, Christopher E. Knoepke, Ryan A. Peterson, Frederick P. Rivara, Ali Rowhani-Rahbar

**Affiliations:** 1 Department of Emergency Medicine, School of Medicine, University of Colorado Anschutz Medical Campus, Aurora, CO USA; 2 Injury and Violence Prevention Center, Colorado School of Public Health, University of Colorado Anschutz Medical Campus, Aurora, CO USA; 3 VA Eastern Colorado Geriatric Research Education and Clinical Center, Denver, CO USA; 4 Department of Epidemiology, Colorado School of Public Health, Aurora, CO USA; 5 Department of Biostatistics & Informatics, Colorado School of Public Health, Aurora, CO USA; 6 Division of Cardiology, School of Medicine, University of Colorado Anschutz Medical Campus, Aurora, CO USA; 7 Adult & Child Consortium for Outcomes Research & Delivery Science, School of Medicine, University of Colorado Anschutz Medical Campus, Aurora, CO USA; 8 Firearm Injury Policy and Research Program, Harborview Injury Prevention and Research Center, University of Washington, Seattle, WA USA; 9 Department of Epidemiology, School of Public Health, University of Washington, Seattle, WA USA

**Keywords:** Firearm, Law enforcement, Suicide, Injury prevention, Storage, Community program

## Abstract

**Background:**

Temporary, voluntary storage of firearms away from the home during times of risk is a recommended strategy for suicide prevention. Law enforcement agencies (LEAs) are often suggested as storage sites, and online maps in Colorado and Washington display LEAs willing to consider storage. Questions remain about the experiences and views of LEAs, including barriers to providing storage.

**Methods:**

LEAs in Colorado and Washington were invited to complete a survey via mail or online from June to July 2021; invitations were sent by email and mail, with telephone calls to non-responders. Survey data were analyzed using descriptive statistics, with testing between states and other subgroups using Fisher’s exact tests.

**Results:**

Overall, 168 LEAs in Colorado (*n* = 91) or Washington (*n* = 77) participated (40% participation rate). Of those, 53% provided temporary, voluntary storage upon request by community members at the time of the survey. More LEAs said they had ever provided storage when the requester was under a court order (74% overall). Over half (60%) of responding LEAs had received at least one storage request in the prior 12 months. Many (41%) said they had declined to return a firearm after temporary storage due to safety concerns. Most LEAs supported engagement in suicide prevention (89%) and provision of community services (77%), but they simultaneously preferred being a storage option of last resort (73%). Factors negatively influencing storage provision included liability and funding concerns.

**Conclusions:**

In Colorado and Washington, half of LEAs currently offer temporary, voluntary firearm storage upon request. While LEAs support suicide prevention and community engagement, broader provision of storage and participation in online maps may be limited by logistic, liability, and financial concerns. Addressing these barriers may facilitate broader suicide prevention efforts.

**Supplementary Information:**

The online version contains supplementary material available at 10.1186/s40621-022-00389-3.

## Background

Suicide remains a leading cause of death in the United States, and firearms are the method used in the majority (53%) of suicides (CDC 2021). “Lethal means safety”—reducing access to firearms and other lethal methods for those at risk of suicide—is an evidence-based, core component of suicide prevention (Yip et al. 2012; Mann et al. 2005). One recommended method for reducing access is moving firearms out of the home for voluntary, temporary storage elsewhere, such as at a firearm retailer or law enforcement agency (LEA).

LEAs may store firearms recovered in criminal cases (e.g., as evidence), by court order (e.g., under a Domestic Violence Restraining Order or Extreme Risk Protection Order), or voluntary request—whereby a community member leaves their firearms in the temporary possession of a LEA for safe storage but is typically able to retrieve their firearms without legal proceedings beyond passing a background check. In a prior survey of LEAs in the eight states in the Mountain West, 74.8% of respondents (including police and sheriff departments) said they had provided temporary firearm storage within the past year (Brooks-Russell et al. 2019; Runyan et al. 2017). LEAs were most likely to provide storage in hypothetical cases where a household member was worried about the mental stability of an adolescent (65% very or somewhat likely to provide storage) or of an adult (63%) (Runyan et al. 2017). Other reasons for providing voluntary storage included an individual wanting to secure guns while traveling (64%) or while having visitors (61%), or an individual wanting to comply with a court order (61%) (Runyan et al. 2017). Generally, LEAs in the survey saw benefits to providing storage, such as supporting the community; identified barriers included lack of space and potential distrust of LEAs as a storage option by the community (Brooks-Russell et al. 2019).

Recently, online maps showing LEAs and firearm retailers willing to consider voluntary storage have been developed. The first map was developed in Colorado in 2019 (Kelly et al. 2020), followed in 2020 in Washington (Washington Firearm Safe Storage Map 2020); while other states have since developed online maps as well, only the Colorado and Washington maps existed when this study was designed. Inclusion of both states allowed for a larger sample and comparisons in two cultural and legislative contexts. Colorado has the 7^th^ highest suicide death rate in the United States, compared to the 26^th^ highest in Washington (21.5 versus 15.2 per 100,000 in 2020) (CDC 2022). However, suicide accounts for the majority of all firearm deaths in both states (71% in Colorado in 2020; 72% in Washington) (CDC 2021). The states are similar in their firearm laws: for example, neither requires purchasing permits, registration, or licensing, but both have Extreme Risk Protection Order laws, require background checks for most transfers, require permits to carry a handgun, and impose penalties for negligent firearm storage (e.g., when a minor could gain access) (McCourt et al. 2017).

While online storage maps resources may help the public find nearby storage options, many questions remain about the experiences of LEAs and retailers in both providing storage and being willing to be listed in an online map. In related qualitative work with potential storage suppliers, we found that LEAs and retailers in Colorado and Washington supported efforts for suicide prevention but had lingering concerns (Betz et al. 2022). These included questions about the logistics and potential liability of providing storage, especially after returning firearms to an owner with possible suicide risk. At the time of the qualitative interviews and the current survey, neither state had liability protection for retailers; in the June 2022, Washington enacted a law with a provision for some civil liability protection for retailers—but not LEAs—who provide voluntary, temporary storage (Washington State Legislature 2022).

Here, we sought to further explore the experiences and views of LEAs across these two states concerning provision of temporary, voluntary firearm storage and participation in online storage maps. Specifically, a better understanding of barriers to facilitators to storage and map participation could directly impact implementation and maintenance of state-wide programs.

## Methods

### Design

Eligible participants were English-speaking individuals associated with a Colorado or Washington State LEA, using a mailing list rented from the National Public Safety Information Bureau (National Public Safety Information Bureau 2022). The contact information for LEAs is updated annually with continuous data verification year-round. Eligible LEAs were municipal law enforcement or county sheriffs; other forms of law enforcement such as campus police, county jails, training facilities, park police and state police were excluded as they are unlikely to have accessible community facilities for temporary firearm storage. We addressed our invitations to the Chief of Police or Sheriff.

Survey invitations were sent by email (when an email address was available) or mail, with options to complete online (via REDCap) or by returning the mailed paper version of the survey from June to July of 2021. Invitations included a cover letter explaining the study, a paper copy of the survey, a stamped return envelope, and a hyperlink for online completion. Non-responders were contacted by up to three emails, three letters, or three phone calls. Paper surveys were entered into the REDCap database by trained study staff. Participants were offered a $50 incentive for completing the survey. This study was deemed exempt by the Colorado Multiple Institutional Review Board and the University of Washington Institutional Review Board.

### Survey instrument

The 34-item survey instrument included questions on: storage experiences, perceived barriers and facilitators to participation in storage maps, policy recommendations, and optimal avenues for public education about out-of-home storage. The survey included both de novo and existing items from a prior survey of storage suppliers conducted in 2016 (Brooks-Russell et al. 2019; Runyan et al. 2017). We pretested the instrument through cognitive interviews with six firearm experts and LEAs in other states (i.e., not in our sample) and through review with our Study Advisory Board, and we subsequently adjusted the instrument as needed to reduce sources of response error.

## Analysis

Survey data were reviewed by Qualtrics and a study team member to assess response completeness and quality. This included excluding surveys with inconsistencies between survey responses, implausible responses, or comments that made participants ineligible (such as they were no longer employed as a LEA). From 420 LEAs invited to participate, 168 consented to the survey and were included in this analysis. RUCA codes, derived from ZIP codes, were used to identify LEAs as urban or rural (Ers et al. 2020). Participant responses were summarized overall and by current storage practice (dichotomized as currently do offer vs do not) with frequencies and percentages; differences between storage groups were tested with Fisher’s exact tests due to small sample sizes in some cells. An alpha level of 0.05 was used for significance testing. All analyses were performed using R Statistical Software (version 4.1.2; R Foundation for Statistical Computing, Vienna, Austria).

## Results

Overall, 168 LEAs in Colorado (*n* = 91; 43% participation) and Washington (*n* = 77; 36% participation) completed the survey (40% overall participation). Most individuals completing the survey on behalf of their LEA identified as non-Hispanic (65%), White (82%), and male (75%), with no differences in race, ethnicity, or gender by state. In both states, the respondent was most often the Chief of Police (57%), followed by Sheriffs (23% in Colorado, 8% in Washington), Deputy Chief of Police (5%), Under-Sheriff (2%), or other administrative role. Among participating LEAs, half (50%) were located in urban areas (37% in Colorado, 64% in Washington); participants and non-participants were similar proportions in urban and rural areas.

Approximately half (53%, *n* = 90) of LEAs said they currently provide temporary, voluntary firearm storage upon request by community members (51% in Colorado, 57% in Washington; *p* = 0.718; Table 1). Slightly more (61%, *n* = 101) LEAs said they had ever offered such storage, even if they were not currently offering storage. A greater proportion of LEAs (74%, *n* = 124) had provided storage when the requester was under a court order to move firearms out of their home; this was less common in Colorado (56%, *n* = 51) than in Washington (95%, *n* = 73; *p* < 0.001).

**Table 1:** Firearm storage experiences of responding law enforcement agencies who have ever provided storage (*n* = 126)

	Overall (*N* = 126)	Colorado (*N* = 63)	Washington (*N* = 63)	*p* value
*Circumstances in which storage provided^*				
Safety concerns/suicide concerns	92 (73.0%)	44 (69.8%)	48 (76.2%)	0.547
Upon request	58 (46.0%)	25 (39.7%)	33 (52.4%)	0.211
Relative passed away	46 (36.5%)	17 (27.0%)	29 (46.0%)	0.041
During addiction, medical or mental health treatment	41 (32.5%)	23 (36.5%)	18 (28.6%)	0.447
Divorce	17 (13.5%)	9 (14.3%)	8 (12.7%)	> 0.999
Prohibited individual is guest in the home	15 (11.9%)	8 (12.7%)	7 (11.1%)	> 0.999
Related to ERPO, court order, or domestic violence	25 (19.8%)	8 (12.7%)	17 (27.0%)	0.073
Travel out of town	8 (6.3%)	2 (3.2%)	6 (9.5%)	0.273
Military deployment	2 (1.6%)	1 (1.6%)	1 (1.6%)	> 0.999
Moving	1 (0.8%)	0 (0.0%)	1 (1.6%)	> 0.999
Other	22 (17.5%)	8 (12.7%)	14 (22.2%)	0.240
*Circumstances in which storage denied^*				
Reason for storing not appropriate	47 (37.3%)	25 (39.7%)	22 (34.9%)	0.713
Not enough storage space	17 (13.5%)	12 (19.0%)	5 (7.9%)	0.116
Not their firearm	14 (11.1%)	10 (15.9%)	4 (6.3%)	0.155
Other	5 (4.0%)	2 (3.2%)	3 (4.8%)	> 0.999
Not applicable	17 (1.0%)	9 (9.9%)	8 (10.4%)	> 0.999
Frequency of providing storage upon request				0.276
For all requests	73 (57.9%)	31 (49.2%)	42 (66.7%)	
For more than half of requests	19 (15.1%)	11 (17.5%)	8 (12.7%)	
For less than half of requests, but at least once	28 (22.2%)	16 (25.4%)	12 (19.0%)	
Storage processes for court-ordered versus voluntary storage				0.477
Handled differently	53 (42.1%)	25 (39.7%)	28 (44.4%)	
Handled the same	57 (45.2%)	27 (42.9%)	30 (47.6%)	
Do not provide both	14 (11.1%)	9 (14.3%)	5 (7.9%)	
Agency has ever declined to return firearm that was being temporarily stored in your facility due to safety concerns				0.016
Yes	51 (40.5%)	19 (30.2%)	32 (50.8%)	
No	69 (54.8%)	42 (66.7%)	27 (42.9%)	

^Multiple responses allowed

Table includes data from responding agencies who reported ever having provided storage (*n* = 128 out of *N* = 168 participants). Responses may not add to 100% due to missing data (not shown if < 5%)

In the past 12 months, over half (60%, *n* = 99) of responding LEAs had received at least one request for storage, less commonly in Colorado (52%, *n* = 47) than in Washington (68%, *n* = 52; *p* = 0.049; Table 1). Among LEAs who had received a request, 55% reported one or two requests, 30% three to nine, and 15% ten or more. In Washington, 21% of LEAs with at least one request reported receiving 10–20 total requests within the past 12 months. LEAs who had received storage requests were asked how they thought requesters had learned about storage options, with multiple responses allowed; most commonly word of mouth (31.1%), the LEA’s website/referral (15.1%), court (8.4%), firearm retailer/range (4.2%), a gun storage map (3.4%), a healthcare provider (2.5%), or an advertisement (1.7%). Many LEAs (41%, *n* = 51) reported having declined to return a firearm after temporary storage due to safety concerns; this was less common in Colorado (30%, *n* = 19) than in Washington (51%, *n* = 32; *p* < 0.05). When asked if they were more or less likely to provide storage related to a court order, compared to a voluntary request, a smaller proportion of LEAs in Colorado than Washington reported that they would be more likely if it were a court order (56.1% vs. 78.0%; *p* < 0.01). The majority (88.0%) of LEAs said that the COVID-19 pandemic had not changed the frequency of requests.

**Fig. 1:**
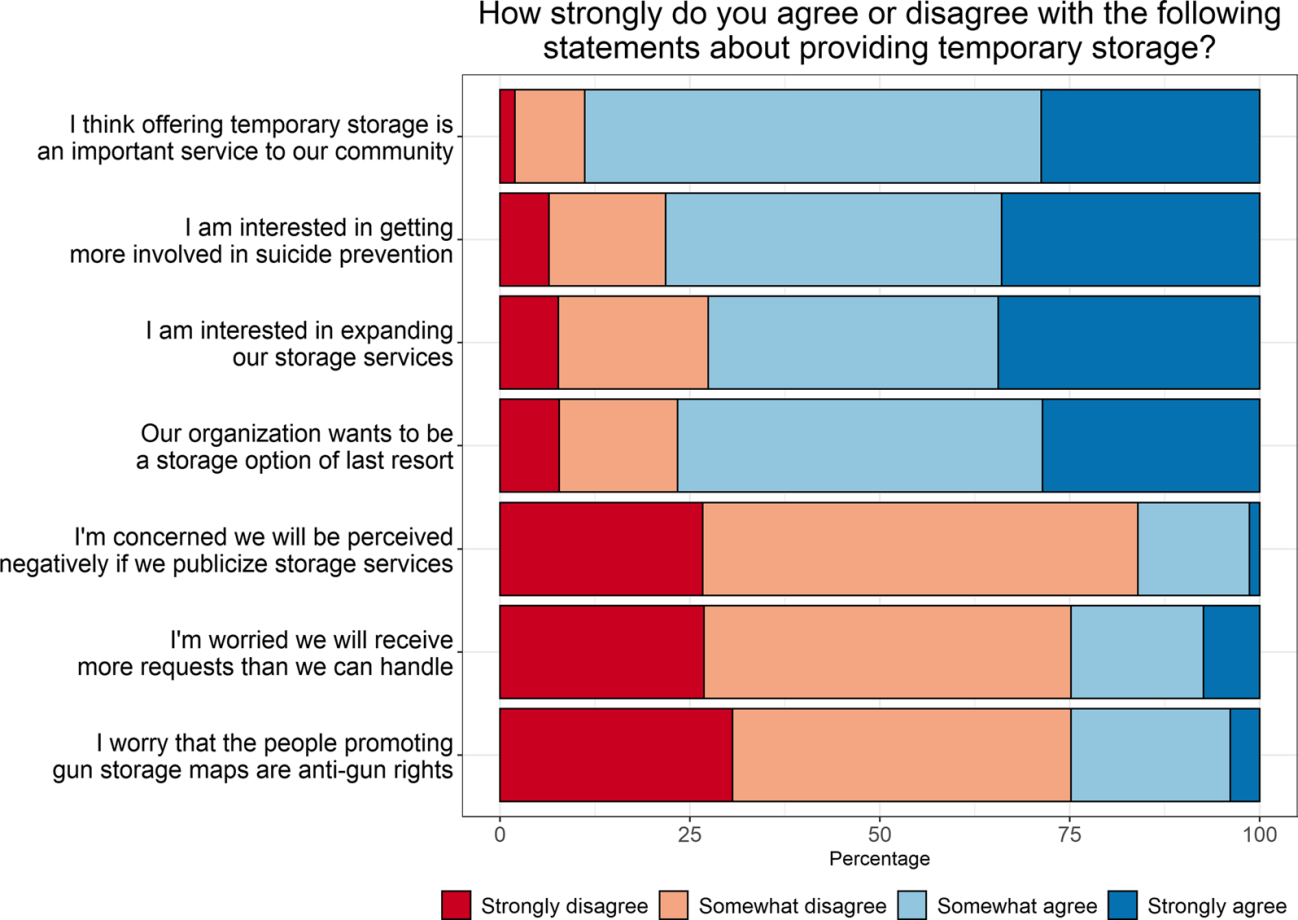
Views on providing temporary storage (*n* = 161). Figure includes data from participants who provided responses to this series of questions (*n* = 161 out of *N* = 168 participants)

**Fig. 2:**
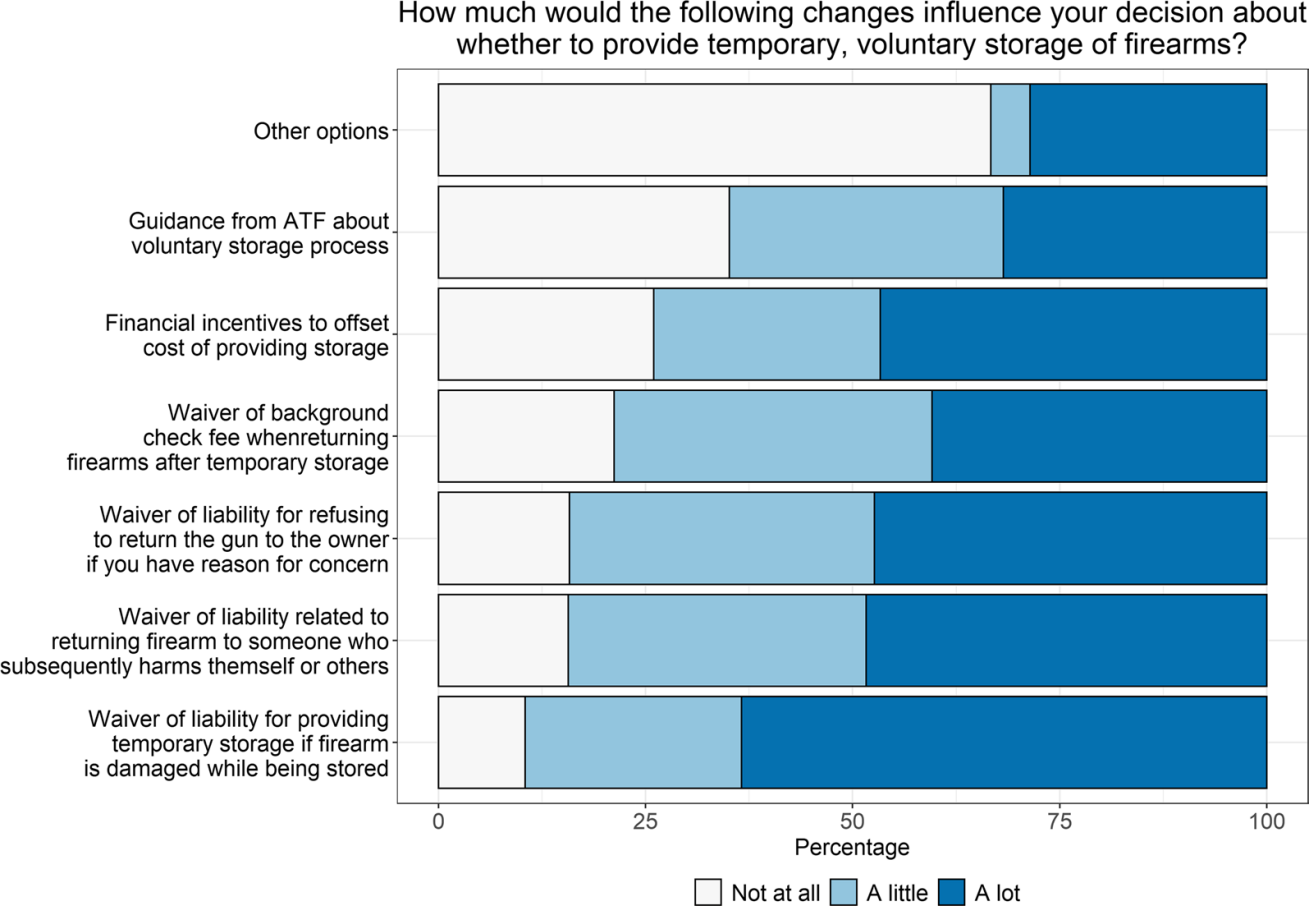
Influences on decision about providing temporary, voluntary firearm storage (*n* = 154). Figure includes data from participants who provided responses to this series of questions (*n* = 154 out of *N* = 168 participants)

When asked about their views on providing temporary storage, a majority of LEAs reported agreeing or strongly agreeing that they wanted to be more involved in suicide prevention (89%) and thought offering temporary storage was an important community service (77%; Fig. 1, Additional file 1: Table 1). However, most LEAs were worried about getting more requests than they could handle (73%) and said their agency preferred to be a storage option of last resort (78%; Fig. 1, Additional file 1: Table 1). When asked about factors that might influence their decision “a lot” to provide temporary, voluntary firearm storage, the greatest proportion of LEAs said availability of a liability waiver related to returning the firearm to someone who subsequently harms themself or others (58%; Fig. 2, Additional file 1: Table 1), followed by liability waivers for damage to firearm during storage (44%) or for refusing to return firearm (43%), and funding to offset storage costs (42%). Greater proportions of LEAs in Washington than in Colorado reported liability waivers as having “a lot” of influence on their decision to provide storage.

**Fig. 3:**
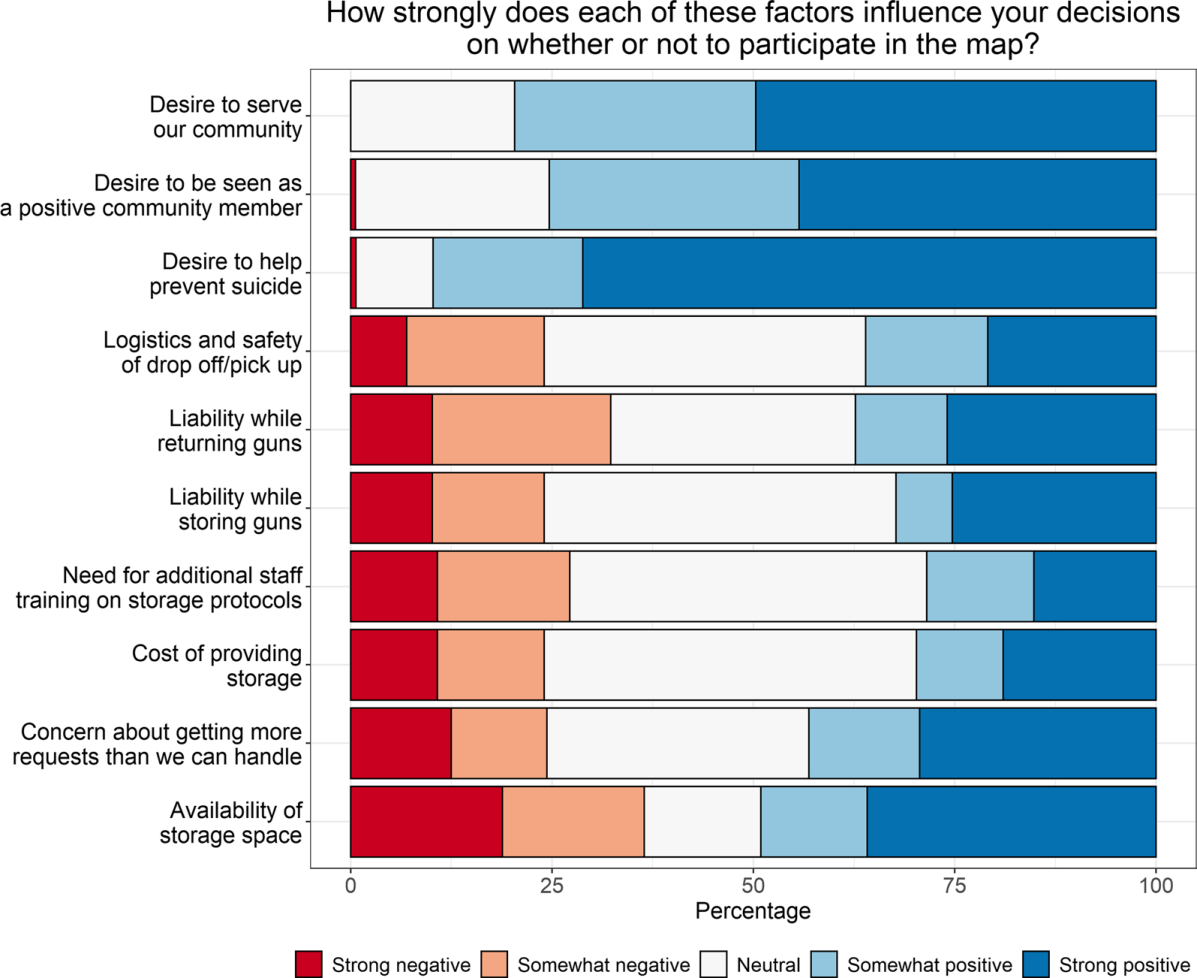
Influences on decision to participate in online map of firearm storage locations (*n* = 160). Figure includes data from participants who provided responses to this series of questions (*n* = 160 out of *N* = 168 participants)

**Fig. 4:**
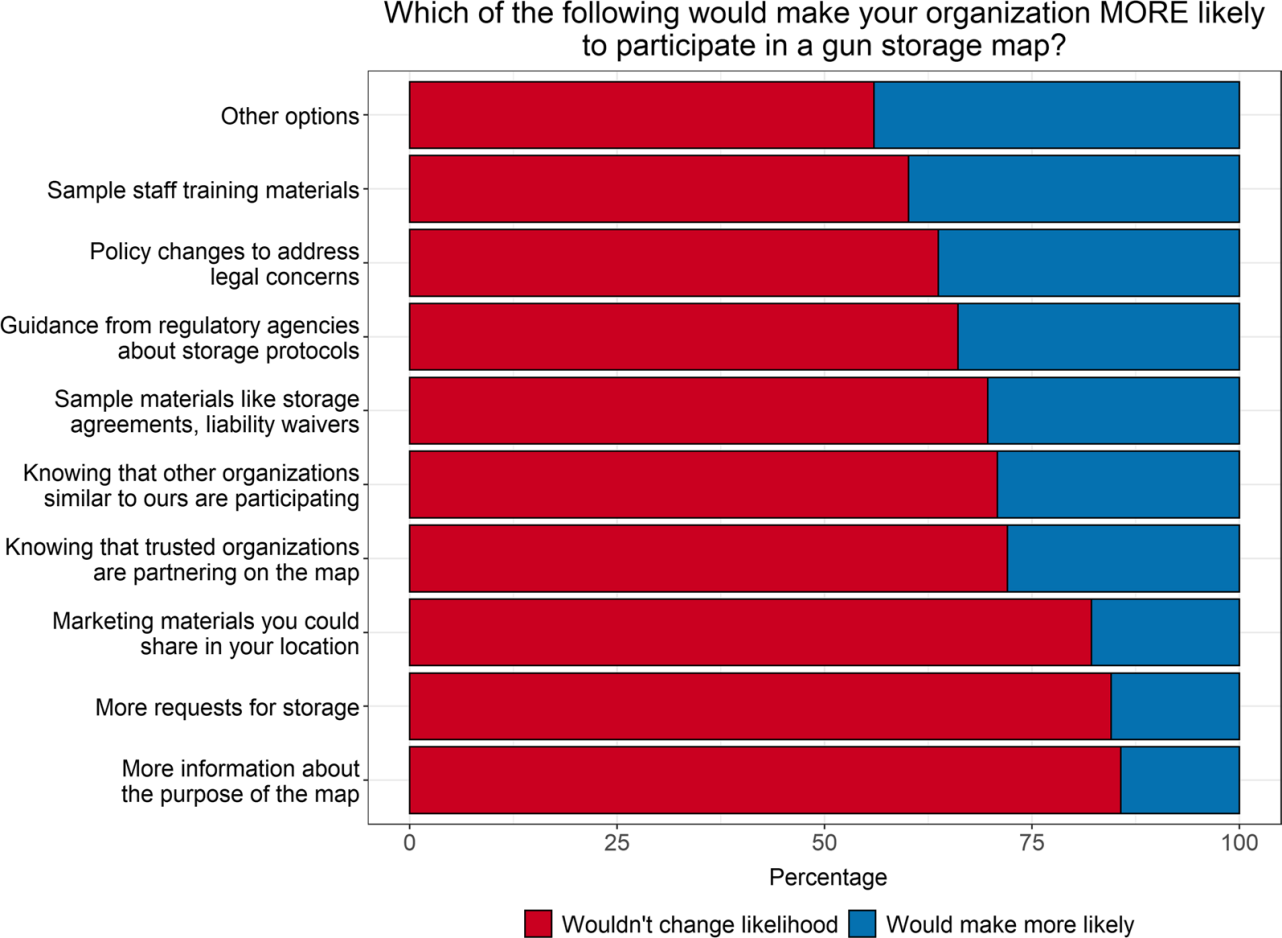
Influences on decision to participate (*n* = 168)

Separate from actual provision of storage, LEAs were also asked about their participation in an online map of potential storage locations. Overall, 29% of LEAs had heard of the maps (37% in Colorado, 18% in Washington; *p* = 0.005), but only 11% (*n* = 19) said that their agency was listed. Among these, most said their agency personnel supported participation completely (53%) or somewhat (32%). These LEAs thought the community probably didn’t know about the LEA’s participation in the map (56%) and said that, since map participation, storage requests had stayed about the same (68%) or increased slightly (21%). When asked about the relative strength of factors influencing the decision to participate in the map, the positive factors identified by the largest proportion of respondents were desires to help prevent suicide (66%), to serve the community (46%), and to be seen as a positive community member (42%; Fig. 3, Additional file 1: Table 1). When asked which factors would make it more likely that the agency would participate in a firearm storage map, the greatest proportion of LEAs reported sample materials (44%), followed by policy changes to address legal concerns (40%), knowing that similar organizations are participating (36%), and more information about the purpose of the map (34%; Fig. 4, Additional file 1: Table 1). In Washington, 43% of LEAs said that knowing that trusted organizations were partnering on the map would influence their own participation, compared to only 20% in Colorado (*p* = 0.001).

When asked about how to share information with individuals or with potential storage locations, LEAs in both Colorado and Washington indicated a range of approaches, ranging from locally-posted flyers to information from trusted organizations to social media (Table 2).

**Table 2:** Views of participating LEAs on the best ways to share information… (*n* = 168)

…in your community about options for voluntary, temporary firearm storage to prevent firearm injury and suicide?*	Overall (*N* = 168)	Colorado (*N* = 91)	Washington (*N* = 77)	*p* value
Information posted in our location (flyers/brochures)	68 (40.5%)	34 (37.4%)	34 (44.2%)	0.431
Information provided at point-of-sale for firearms	37 (22.0%)	19 (20.9%)	18 (23.4%)	0.713
TV/Radio	20 (11.9%)	3 (3.3%)	17 (22.1%)	< 0.001
Internet	47 (28.0%)	24 (26.4%)	23 (29.9%)	0.73
Social media	114 (67.9%)	57 (62.6%)	57 (74.0%)	0.137
In partnership with organizations like the Fraternal Order of Police'	17 (10.1%)	10 (11.0%)	7 (9.1%)	0.8
Sharing information via health care and mental health providers	88 (52.4%)	43 (47.3%)	45 (58.4%)	0.165
Other	8 (4.8%)	3 (3.3%)	5 (6.5%)	0.472
…with law enforcement agencies about participating in gun storage maps*				
Information from trusted national organizations	61 (36.3%)	33 (36.3%)	28 (36.4%)	> 0.999
Information from trusted statewide organizations	103 (61.3%)	51 (56.0%)	52 (67.5%)	0.153
Information from community organizations	41 (24.4%)	26 (28.6%)	15 (19.5%)	0.208
Personal outreach	38 (22.6%)	22 (24.2%)	16 (20.8%)	0.712
Mailings	12 (7.1%)	6 (6.6%)	6 (7.8%)	0.773
Email contact	42 (25.0%)	20 (22.0%)	22 (28.6%)	0.373
Phone contact	7 (4.2%)	4 (4.4%)	3 (3.9%)	> 0.999
Other	9 (5.4%)	2 (2.2%)	7 (9.1%)	0.081

*Up to three choices allowed

## Discussion

In Colorado and Washington, slightly more than half of LEAs currently provide temporary, voluntary firearm storage. Our survey findings build on prior work to expand our understanding of the experiences of and influences on LEAs in providing storage to community members and in being identified as storage locations on publicly available online maps. In line with prior work (Brooks-Russell et al. 2019; Runyan et al. 2017; Betz et al. 2022), LEAs expressed strong interest in helping to prevent suicide and provide services to their community, yet were also concerned about logistical and liability issues. Our findings have direct implications for action, including needed policy changes and approaches for engagement toward suicide prevention.

Among responding LEAs, 78% said that their agency would want to be the storage option of last resort, citing concerns around liability, space, and other logistics. Smaller agencies may have limited physical space or devices to ensure secure storage, and all agencies need to develop systems by which to differentiate firearms stored voluntarily from those stored as evidence in criminal proceedings. Financial concerns also relate to the fee for the background required when the firearm is returned, as some agencies may not be able to pass this fee on to the individual; indeed, nearly a third of agencies said that waiving this background check fee for law enforcement would make them a lot more likely to provide storage. Liability, especially after returning firearms, has been identified as a concern for LEAs as well as for firearm retailers or ranges who provide storage (Runyan et al. 2017; Betz et al. 2022); reducing such liability without dedicated legislation may unfortunately be difficult. Actions to encourage LEA provision of storage that would not require legislation include funding to support storage (e.g., for updating physical spaces or purchasing locking devices) or storage processes, as well as clarification of transfer policies (Betz et al. 2022). LEAs in this survey also identified a desire for sample policies or protocols; these might be shared informally among agencies, or models could be developed and disseminated by larger organizations.

While this study highlights the many challenges to LEAs in providing storage, another clear finding was the desire of LEAs to help prevent suicide and serve their community, despite only 11% reporting being listed on the map. Equally strong was a desire to be *seen* as a positive community member, including through efforts to operationalize that desire into internal policy and action (i.e., providing voluntary storage as a normal matter of course). In other areas where uptake of community-level interventions are challenging (including LEA firearm policy contexts) (Schroeder et al. 2022; Pear et al. 2021), the need to develop or use existing networks of interagency communication was seen as necessary to these efforts. In this way, LEAs could offer each other advise about how to support voluntary, temporary storage programs—logistically, politically, with sample policy templates, and otherwise—as well as provide gentle “peer pressure” for agencies who have yet to develop storage programs. A “cosmopolitan” approach to information and support sharing simultaneously engenders community within and among participating agencies and organically provides technical assistance, creating a social process supportive of uptake at the organizational level (Damschroder et al. 2009). Institutional theory posits that change can come through new rules or coercive pressures, through “mimetic pressures” to copy successful strategies as they become more widely adopted, or normative processes that have been documented to lead to diffusion of innovation in law enforcement agencies (Burruss and Giblin 2014).

Study limitations include that our study involved LEAs in only two states, so results may not generalize to other states, including states with different firearm-related laws. Responding agencies may be more supportive of suicide prevention and temporary storage than non-respondents. A larger study in more states would allow more detailed comparisons or analyses across subgroups; our sample size limited such analyses. A larger study could also allow further exploration of potential differences between types of LEAs, such as police departments versus sheriff offices. We did not include tribal law enforcement agencies due to logistic complexities, including the need for institutional review board review by each tribe. Our survey questions focused primarily on suicide or court-ordered, but community members may also seek out-of-home firearm storage for other reasons (e.g., travel, visitors, or concern that a family member is at risk of hurting others), and further work might explore LEA views on these situations.

## Conclusions

Law enforcement organizations across Colorado and Washington State largely support suicide prevention efforts, including a desire to provide voluntary, temporary firearm storage for individuals or families experiencing crisis. Barriers to more widespread participation remain, however, including logistic, legal, and policy issues. Opportunities exist to promote community-based firearm injury and suicide prevention, some of which are actionable in the short term. Future work should focus on policy and legal remedies to improve adoption of voluntary storage programs nationally.

## Supplementary Information

**Additional file 1**. **Supplemental Table 1**. Data from Figures 1 to 4. Differences by state are tested with Fisher’s exact tests, due to small sample sizes in some cells.

## Data Availability

A deidentified version of the dataset used for this study is available from the corresponding author upon reasonable request.

## References

[CR1] Brooks-Russell A, Runyan C, Betz ME, Tung G, Brandspigel S, Novins DK (2019). Law enforcement agencies’ perceptions of the benefits of and barriers to temporary firearm storage to prevent suicide. Am J Public Health.

[CR2] Burruss GW, Giblin MJ (2014). Modeling isomorphism on policing innovation: the role of institutional pressures in adopting community-oriented policing. Crime Delinq.

[CR3] Damschroder LJ, Aron DC, Keith RE, Kirsh SR, Alexander JA, Lowery JC (2009). Fostering implementation of health services research findings into practice: a consolidated framework for advancing implementation science. Implement Sci.

[CR4] Kelly T, Brandspigel S, Polzer E, Betz ME (2020). Firearm storage maps: a pragmatic approach to reduce firearm suicide during times of risk. Ann Internal Med.

[CR5] Mann JJ, Apter A, Bertolote J, Beautrais A, Currier D, Haas A (2005). Suicide prevention strategies: a systematic review. JAMA.

[CR6] McCourt AD, Vernick JS, Betz ME, Brandspigel S, Runyan CW (2017). Temporary transfer of firearms from the home to prevent suicide: legal obstacles and recommendations. JAMA Intern Med.

[CR7] Pear VA, Schleimer JP, Tomsich E, Pallin R, Charbonneau A, Wintemute GJ (2021). Implementation and perceived effectiveness of gun violence restraining orders in California: a qualitative evaluation. PLoS ONE.

[CR8] Runyan CW, Brooks-Russell A, Brandspigel S, Betz ME, Tung G, Novins D (2017). Law enforcement and gun retailers as partners for safely storing guns to prevent suicide: a study in 8 Mountain West States. Am J Public Health.

[CR9] Schroeder D, Luig T, Finch TL, Beesoon S, Campbell-Scherer DL (2022). Understanding implementation context and social processes through integrating Normalization Process Theory (NPT) and the Consolidated Framework for Implementation Research (CFIR). Implement Sci Commun.

[CR10] Yip PS, Caine E, Yousuf S, Chang SS, Wu KC, Chen YY (2012). Means restriction for suicide prevention. Lancet.

[CR11] Betz ME, Rooney LA, Barnard LM, Siry-Bove BJ, Brandspigel S, McCarthy M, Simeon K, Meador L, Rivara FP, Rowhani-Rahbar A, Knoepke CE (2022). Voluntary, temporary, out-of-home firearm storage: A qualitative study of stakeholder views. Suicide Life Threat Behav..

[CR12] National Public Safety Information Bureau [Internet]. 2022 [cited 2022 Apr 29]. Available from: https://www.safetysource.com/.

[CR13] CDC. Suicide Mortality by State [Internet]. Centers for Disease Control and Prevention; 2022 [cited 2022 Jun 23]. Available from: https://www.cdc.gov/nchs/pressroom/sosmap/suicide-mortality/suicide.htm.

[CR14] CDC. Web-based Injury Statistics Query and Reporting System (WISQARS) [Internet]. Centers for Disease Control and Prevention, National Center for Injury Prevention and Control; 2021. Available from: http://www.cdc.gov/injury/wisqars/index.html.

[CR15] USDA ERS - Rural-Urban Commuting Area Codes [Internet]. 2020 [cited 2022 Jan 28]. Available from: https://www.ers.usda.gov/data-products/rural-urban-commuting-area-codes/.

[CR16] Washington State Legislature [Internet]. [cited 2022 Jun 23]. Available from: https://apps.leg.wa.gov/billsummary?year=2022&billnumber=1181&initiative=false.

[CR17] Washington Firearm Safe Storage Map [Internet]. 2020. Available from: http://depts.washington.edu/hiprc/firearm-storage-wa/.

