# Supplementary material for: Voluntary, temporary out-of-home firearm storage: a survey of law enforcement agencies in two states
Source: Inj Epidemiol. 2022 Jul 21;9:24. doi: 10.1186/s40621-022-00389-3 (PMC9302866; doi:10.1186/s40621-022-00389-3)
Supplement: Supplementary file 1 — Additional file 1. Supplemental Table 1. Data from Figures 1 to 4. Differences by state are tested with Fisher’s exact tests, due to small sample sizes in some cells. [file 40621_2022_389_MOESM1_ESM.docx]

**Supplemental Table 1.** Data from Figures 1 to 4. Differences by state are tested with Fisher’s exact tests, due to small sample sizes in some cells.

|  | **Overall**  (N = 168) | **Colorado**  (N = 91) | **Washington**  (N = 77) | **p value** |
| --- | --- | --- | --- | --- |
| **Views on providing temporary storage (Figure 1)** |  |  |  |  |
| I think offering temporary storage is an important service to our community |  |  |  | 0.983 |
| Strongly agree | 44 (27.2%) | 22 (25.6%) | 22 (28.9%) |  |
| Somewhat agree | 74 (45.7%) | 41 (47.7%) | 33 (43.4%) |  |
| Somewhat disagree | 24 (14.8%) | 13 (15.1%) | 11 (14.5%) |  |
| Strongly disagree | 12 (7.4%) | 6 (7.0%) | 6 (7.9%) |  |
| Missing | N = 8 | N = 4 | N = 4 |  |
| I am interested in getting more involved in suicide prevention |  |  |  | 0.182 |
| Strongly agree | 44 (27.2%) | 30 (34.9%) | 14 (18.4%) |  |
| Somewhat agree | 92 (56.8%) | 44 (51.2%) | 48 (63.2%) |  |
| Somewhat disagree | 14 (8.6%) | 7 (8.1%) | 7 (9.2%) |  |
| Strongly disagree | 3 (1.9%) | 1 (1.2%) | 2 (2.6%) |  |
| Missing | N = 9 | N = 4 | N = 5 |  |
| I am interested in expanding our storage services |  |  |  | 0.733 |
| Strongly agree | 6 (3.7%) | 3 (3.5%) | 3 (3.9%) |  |
| Somewhat agree | 33 (20.4%) | 15 (17.4%) | 18 (23.7%) |  |
| Somewhat disagree | 70 (43.2%) | 36 (41.9%) | 34 (44.7%) |  |
| Strongly disagree | 48 (29.6%) | 29 (33.7%) | 19 (25.0%) |  |
| Missing | N = 5 | N = 3 | N = 2 |  |
| Our organization wants to be a storage option of last resort |  |  |  | 0.439 |
| Strongly agree | 53 (32.7%) | 33 (38.4%) | 20 (26.3%) |  |
| Somewhat agree | 69 (42.6%) | 33 (38.4%) | 36 (47.4%) |  |
| Somewhat disagree | 24 (14.8%) | 12 (14.0%) | 12 (15.8%) |  |
| Strongly disagree | 10 (6.2%) | 4 (4.7%) | 6 (7.9%) |  |
| Missing | N = 6 | N = 4 | N = 2 |  |
| I'm concerned we will be perceived negatively if we publicize storage services |  |  |  | 0.533 |
| Strongly agree | 2 (1.2%) | 2 (2.3%) | 0 (0.0%) |  |
| Somewhat agree | 22 (13.6%) | 13 (15.1%) | 9 (11.8%) |  |
| Somewhat disagree | 86 (53.1%) | 42 (48.8%) | 44 (57.9%) |  |
| Strongly disagree | 40 (24.7%) | 21 (24.4%) | 19 (25.0%) |  |
| Missing | N = 12 | N = 8 | N = 4 |  |
| I'm worried we will receive more requests than we can handle |  |  |  | 0.530 |
| Strongly agree | 54 (33.3%) | 29 (33.7%) | 25 (32.9%) |  |
| Somewhat agree | 60 (37.0%) | 28 (32.6%) | 32 (42.1%) |  |
| Somewhat disagree | 31 (19.1%) | 17 (19.8%) | 14 (18.4%) |  |
| Strongly disagree | 12 (7.4%) | 8 (9.3%) | 4 (5.3%) |  |
| Missing | N = 5 | N = 4 | N = 1 |  |
| I worry that the people promoting gun storage maps are anti-gun rights |  |  |  | 0.399 |
| Strongly agree | 11 (6.8%) | 7 (8.1%) | 4 (5.3%) |  |
| Somewhat agree | 26 (16.0%) | 13 (15.1%) | 13 (17.1%) |  |
| Somewhat disagree | 72 (44.4%) | 35 (40.7%) | 37 (48.7%) |  |
| Strongly disagree | 40 (24.7%) | 21 (24.4%) | 19 (25.0%) |  |
| Missing |  |  |  |  |
| **Influences on decision about providing temporary, voluntary firearm storage (Figure 2)** |  |  |  |  |
| Guidance from ATF about voluntary storage process |  |  |  | 0.637 |
| A lot | 61 (39.6%) | 31 (39.2%) | 30 (40.0%) |  |
| A little | 58 (37.7%) | 27 (34.2%) | 31 (41.3%) |  |
| Not at all | 32 (20.8%) | 19 (24.1%) | 13 (17.3%) |  |
| Missing | N = 3 | N = 2 | N = 1 |  |
| Financial incentives to offset cost of providing storage |  |  |  | 0.315 |
| A lot | 70 (45.5%) | 32 (40.5%) | 38 (50.7%) |  |
| A little | 41 (26.6%) | 20 (25.3%) | 21 (28.0%) |  |
| Not at all | 39 (25.3%) | 25 (31.6%) | 14 (18.7%) |  |
| Missing | N = 4 | N = 2 | N = 2 |  |
| Waiver of background check fee when returning firearms after temporary storage |  |  |  | 0.003 |
| A lot | 48 (31.2%) | 15 (19.0%) | 33 (44.0%) |  |
| A little | 50 (32.5%) | 29 (36.7%) | 21 (28.0%) |  |
| Not at all | 53 (34.4%) | 34 (43.0%) | 19 (25.3%) |  |
| Missing | N = 3 | N = 1 | N = 2 |  |
| Waiver of liability for refusing to return the gun to the owner if you have reason for concern |  |  |  | 0.531 |
| A lot | 72 (46.8%) | 33 (41.8%) | 39 (52.0%) |  |
| A little | 56 (36.4%) | 30 (38.0%) | 26 (34.7%) |  |
| Not at all | 24 (15.6%) | 15 (19.0%) | 9 (12.0%) |  |
| Missing | N = 2 | N = 1 | N = 1 |  |
| Waiver of liability related to returning the firearm to someone who subsequently harms themself or others |  |  |  | 0.156 |
| A lot | 97 (63.0%) | 44 (55.7%) | 53 (70.7%) |  |
| A little | 40 (26.0%) | 23 (29.1%) | 17 (22.7%) |  |
| Not at all | 16 (10.4%) | 11 (13.9%) | 5 (6.7%) |  |
| Missing | N = 1 | N = 1 | N = 0 |  |
| Waiver of liability for providing temporary storage if the firearm is damaged while being stored |  |  |  | 0.041 |
| A lot | 74 (48.1%) | 34 (43.0%) | 40 (53.3%) |  |
| A little | 55 (35.7%) | 27 (34.2%) | 28 (37.3%) |  |
| Not at all | 24 (15.6%) | 18 (22.8%) | 6 (8.0%) |  |
| Missing | N = 1 | N = 0 | N = 1 |  |
| Other (select any option and a text box will appear) |  |  |  | 0.898 |
| A lot | 6 (3.9%) | 3 (3.8%) | 3 (4.0%) |  |
| A little | 1 (0.6%) | 0 (0.0%) | 1 (1.3%) |  |
| Not at all | 14 (9.1%) | 8 (10.1%) | 6 (8.0%) |  |
| Missing | N = 133 | N = 68 | N = 65 |  |
| **Influences on decision to participate in online map of firearm storage locations (Figure 3)** |  |  |  |  |
| Desire to serve our community |  |  |  | 0.623 |
| Strong positive | 78 (48.8%) | 46 (52.3%) | 32 (44.4%) |  |
| Somewhat positive | 47 (29.4%) | 23 (26.1%) | 24 (33.3%) |  |
| Neutral | 32 (20.0%) | 18 (20.5%) | 14 (19.4%) |  |
| Somewhat negative | 0 (0.0%) | 0 (0.0%) | 0 (0.0%) |  |
| Strong negative | 0 (0.0%) | 0 (0.0%) | 0 (0.0%) |  |
| Missing | N = 3 | N = 1 | N = 2 |  |
| Desire to be seen as positive community member |  |  |  | 0.752 |
| Strong positive | 70 (43.8%) | 42 (47.7%) | 28 (38.9%) |  |
| Somewhat positive | 49 (30.6%) | 25 (28.4%) | 24 (33.3%) |  |
| Neutral | 38 (23.8%) | 19 (21.6%) | 19 (26.4%) |  |
| Somewhat negative | 0 (0.0%) | 0 (0.0%) | 0 (0.0%) |  |
| Strong negative | 1 (0.6%) | 1 (1.1%) | 0 (0.0%) |  |
| Missing | N = 2 | N = 1 | N = 1 |  |
| Desire to help prevent suicide |  |  |  | 0.122 |
| Strong positive | 111 (69.4%) | 66 (75.0%) | 45 (62.5%) |  |
| Somewhat positive | 29 (18.1%) | 11 (12.5%) | 18 (25.0%) |  |
| Neutral | 15 (9.4%) | 9 (10.2%) | 6 (8.3%) |  |
| Somewhat negative | 0 (0.0%) | 0 (0.0%) | 0 (0.0%) |  |
| Strong negative | 1 (0.6%) | 1 (1.1%) | 0 (0.0%) |  |
| Missing | N = 4 | N = 1 | N = 3 |  |
| Logistics and safety of drop off/pick up |  |  |  | 0.530 |
| Strong positive | 33 (20.6%) | 15 (17.0%) | 18 (25.0%) |  |
| Somewhat positive | 24 (15.0%) | 12 (13.6%) | 12 (16.7%) |  |
| Neutral | 63 (39.4%) | 36 (40.9%) | 27 (37.5%) |  |
| Somewhat negative | 27 (16.9%) | 15 (17.0%) | 12 (16.7%) |  |
| Strong negative | 11 (6.9%) | 8 (9.1%) | 3 (4.2%) |  |
| Missing | N = 2 | N = 2 | N = 0 |  |
| Liability when returning guns |  |  |  | 0.163 |
| Strong positive | 41 (25.6%) | 18 (20.5%) | 23 (31.9%) |  |
| Somewhat positive | 18 (11.2%) | 12 (13.6%) | 6 (8.3%) |  |
| Neutral | 48 (30.0%) | 29 (33.0%) | 19 (26.4%) |  |
| Somewhat negative | 35 (21.9%) | 16 (18.2%) | 19 (26.4%) |  |
| Strong negative | 16 (10.0%) | 12 (13.6%) | 4 (5.6%) |  |
| Missing | N = 2 | N = 1 | N = 1 |  |
| Liability while storing guns |  |  |  | 0.282 |
| Strong positive | 40 (25.0%) | 17 (19.3%) | 23 (31.9%) |  |
| Somewhat positive | 11 (6.9%) | 5 (5.7%) | 6 (8.3%) |  |
| Neutral | 69 (43.1%) | 39 (44.3%) | 30 (41.7%) |  |
| Somewhat negative | 22 (13.8%) | 14 (15.9%) | 8 (11.1%) |  |
| Strong negative | 16 (10.0%) | 11 (12.5%) | 5 (6.9%) |  |
| Missing | N = 2 | N = 2 | N = 0 |  |
| Need for additional staff training on storage protocols |  |  |  | 0.048 |
| Strong positive | 24 (15.0%) | 14 (15.9%) | 10 (13.9%) |  |
| Somewhat positive | 21 (13.1%) | 6 (6.8%) | 15 (20.8%) |  |
| Neutral | 70 (43.8%) | 37 (42.0%) | 33 (45.8%) |  |
| Somewhat negative | 26 (16.2%) | 17 (19.3%) | 9 (12.5%) |  |
| Strong negative | 17 (10.6%) | 13 (14.8%) | 4 (5.6%) |  |
| Missing | N = 2 | N = 1 | N = 1 |  |
| Concern about getting more requests than we can handle |  |  |  | 0.010 |
| Strong positive | 47 (29.4%) | 20 (22.7%) | 27 (37.5%) |  |
| Somewhat positive | 22 (13.8%) | 8 (9.1%) | 14 (19.4%) |  |
| Neutral | 52 (32.5%) | 38 (43.2%) | 14 (19.4%) |  |
| Somewhat negative | 19 (11.9%) | 10 (11.4%) | 9 (12.5%) |  |
| Strong negative | 20 (12.5%) | 12 (13.6%) | 8 (11.1%) |  |
| Missing | N = 0 | N = 0 | N = 0 |  |
| Cost of providing storage |  |  |  | 0.299 |
| Strong positive | 30 (18.8%) | 14 (15.9%) | 16 (22.2%) |  |
| Somewhat positive | 17 (10.6%) | 6 (6.8%) | 11 (15.3%) |  |
| Neutral | 73 (45.6%) | 42 (47.7%) | 31 (43.1%) |  |
| Somewhat negative | 21 (13.1%) | 13 (14.8%) | 8 (11.1%) |  |
| Strong negative | 17 (10.6%) | 12 (13.6%) | 5 (6.9%) |  |
| Missing | N = 2 | N = 1 | N = 1 |  |
| Availability of storage space |  |  |  | 0.226 |
| Strong positive | 57 (35.6%) | 31 (35.2%) | 26 (36.1%) |  |
| Somewhat positive | 21 (13.1%) | 8 (9.1%) | 13 (18.1%) |  |
| Neutral | 23 (14.4%) | 11 (12.5%) | 12 (16.7%) |  |
| Somewhat negative | 28 (17.5%) | 18 (20.5%) | 10 (13.9%) |  |
| Strong negative | 30 (18.8%) | 20 (22.7%) | 10 (13.9%) |  |
| Missing | N = 1 | N = 0 | N = 1 |  |
| **Influences on decision to participate (Figure 4)** |  |  |  |  |
| Sample staff training materials | 49 (29.2%) | 26 (28.6%) | 23 (29.9%) | 0.866 |
| Policy changes to address legal concerns | 67 (39.9%) | 36 (39.6%) | 31 (40.3%) | 1 |
| Guidance from regulatory agencies about storage protocols | 47 (28.0%) | 25 (27.5%) | 22 (28.6%) | 1 |
| Sample materials like storage agreements, liability waivers | 74 (44.0%) | 32 (35.2%) | 42 (54.5%) | 0.013 |
| Knowing that other organizations similar to ours are participating | 61 (36.3%) | 30 (33.0%) | 31 (40.3%) | 0.339 |
| Knowing that trusted organizations are partnering on the map | 51 (30.4%) | 18 (19.8%) | 33 (42.9%) | 0.001 |
| Marketing materials you could share in your location | 30 (17.9%) | 15 (16.5%) | 15 (19.5%) | 0.688 |
| More requests for storage | 26 (15.5%) | 12 (13.2%) | 14 (18.2%) | 0.399 |
| More information about the purpose of the map | 57 (33.9%) | 25 (27.5%) | 32 (41.6%) | 0.072 |
| Other | 24 (14.3%) | 16 (17.6%) | 8 (10.4%) | 0.268 |
